# L-Glucose: Another Path to Cancer Cells

**DOI:** 10.3390/cancers12040850

**Published:** 2020-04-01

**Authors:** Koki Ono, Shota Takigawa, Katsuya Yamada

**Affiliations:** 1 Department of Physiology, Hirosaki University Graduate School of Medicine, 5 Zaifu-cho, Hirosaki, Aomori 036-8562, Japan; h16gm101@hirosaki-u.ac.jp; 2 Department of Biochemistry and Molecular Biology, Faculty of Agriculture and Life Science, Hirosaki University, 3 Bunkyo-cho, Hirosaki, Aomori 036-8561, Japan; d8zpf9@yahoo.co.jp

**Keywords:** Warburg effect, mitochondria, metabolism, cancer, tumor, heterogeneity, glucose, transporter, GLUT, l-glucose, 2-NBDLG, 2-NBDG, imaging, fluorescence, probe, channel

## Abstract

Cancerous tumors comprise cells showing metabolic heterogeneity. Among numerous efforts to understand this property, little attention has been paid to the possibility that cancer cells take up and utilize otherwise unusable substrates as fuel. Here we discuss this issue by focusing on l-glucose, the mirror image isomer of naturally occurring d-glucose; l-glucose is an unmetabolizable sugar except in some bacteria. By combining relatively small fluorophores with l-glucose, we generated fluorescence-emitting l-glucose tracers (fLGs). To our surprise, 2-NBDLG, one of these fLGs, which we thought to be merely a control substrate for the fluorescent d-glucose tracer 2-NBDG, was specifically taken up into tumor cell aggregates (spheroids) that exhibited nuclear heterogeneity, a major cytological feature of malignancy in cancer diagnosis. Changes in mitochondrial activity were also associated with the spheroids taking up fLG. To better understand these phenomena, we review here the Warburg effect as well as key studies regarding glucose uptake. We also discuss tumor heterogeneity involving aberrant uptake of glucose and mitochondrial changes based on the data obtained by fLG. We then consider the use of fLGs as novel markers for visualization and characterization of malignant tumor cells.

## 1. Introduction

Multicellular organisms are composed of cells assembled three-dimensionally; when packed tightly together, these cells have better chance to tolerate environmental changes than those in unicellular organisms, especially in harsh conditions. Indeed, multicellular aggregates are often detected in patients with high-grade cancers such as advanced ovarian cancer, in their ascites (fluid in the abdominal cavity) [1,2], in which the availability of oxygen is very limited [3]. These cell aggregates (spheroidal, three-dimensionally accumulated tumor cells) are more resistant to hyperthermia, chemotherapeutic drugs and radiation compared to cells proliferated two-dimensionally [4,5,6,7]. 

However, there are biological pros and cons to this spatial arrangement. When equally energy-demanding cells are accumulated tightly and three-dimensionally, the deeper the distance from the surface, the more difficult it is for the cells to get nutrients/oxygen and to excrete waste products [8,9,10]. How can organisms reconcile these opposing needs and survive the worst situations? A possibility would be to exchange fuels and metabolites intercellularly through gap junctions, channels, and transporters [5,11,12,13] and/or to acquire angiogenesis [14,15,16]. Another way might be the cells themselves to change their positions like Emperor Penguins huddling together in tightly-packed masses to endure extreme cold **[17,18]**. 

Another solution at a more fundamental level would be to change cell metabolism or reduce energy requirements. Over a half century ago, the German physiologist Otto Warburg investigated cancer cells in mouse ascites from a thermo-energetic perspective [19]. These ascites cells were not transferred to Ringer’s solution but were maintained in ascites serum with glucose and bicarbonate added. Warburg mentions “the cancer cells can obtain approximately the same amount of energy from fermentation as from respiration, whereas the normal body cells obtain much more energy from respiration than from fermentation”. He further stated “cancer cells require much less energy to keep them alive than they do for growth. In this they resemble other lower cells, such as yeast cells, which remain alive for a long time in densely packed packets—almost without respiration and fermentation.” [19]. This metabolic flexibility or plasticity of cancer cells has attracted great attention as one of the major findings in cancer-related fields [20,21,22]. 

d-glucose is an energy and/or carbon source for nearly all prokaryotes and eukaryotes, but l-glucose is not [23]. Is this because a specific mechanism to take up l-glucose is lacking in ordinary cells? As described in the literature, l-glucose has been used only as an unmetabolizable control substrate against the essential nutrient d-glucose [24]. How then do we understand that some bacteria in soil can take up and catabolize l-glucose [25,26,27]. In the first few sections, we review studies of the glucose uptake system focusing on nomenclature, molecular identity, pharmacology, and physiology, and particularly on the relevance to cancer. In the following sections we discuss the possibility that some mammalian tumor cells may deviate from stereo-preference in glucose uptake based on our single-cell analyses of mouse insulinoma using fluorescence-emitting l- and d-glucose tracers. From related changes detected in mitochondria of these cells that were unexpected, we discuss the diversity in malignant tumor cells from the aspect of glucose uptake as well as metabolism.

## 2. Two Distinct Mechanisms of d-glucose Uptake through the Plasma Membrane of Mammalian Cells

d-glucose is the minimum building block of starch, and is the most common monosaccharide in Nature. Most if not all organisms take extracellular d-glucose into cells though specific membrane-spanning machinery [28,29] such as the glucose transporters and the phosphotransferase system in mammals [30,31,32] and bacteria [33,34], respectively.

In mammals, two distinct glucose transport mechanisms have been extensively investigated: facilitated diffusion [30] and Na^+^/sugar cotransport [32]. In its original formulation, “facilitated diffusion” implies that “net transport always occurs in the direction of high to low sugar concentration, and not by a simple diffusion” [30]. By contrast, in Na^+^/sugar cotransport, “The sugar could be absorbed uphill against its concentration gradient” depending on the electrochemical gradient of the sodium ion across the plasma membrane [32]. Alternatively, glucose transport via Na^+^/sugar cotransport cannot occur when the concentration of sodium ions in the extracellular space is identical to that in the intracellular space [35]. 

In this review, we use “facilitated diffusion” in the narrowest sense to discriminate it from Na^+^/sugar cotransport. It follows the concentration gradient of glucose but is not a simple diffusion across lipid bilayers. The concept “facilitated” diffusion, or “not by a simple diffusion”, may have come from the observation that the uptake of extracellular glucose into human erythrocytes was accelerated by the intracellular d-glucose [36]. More precisely, this effect, termed the “trans-effect,” is “the effect of the presence of sugar on one side of the membrane on the rate of unidirectional transport of sugar from the opposite side of the membrane” [30]. Although not all transport exhibits the trans-effect, it would be difficult to explain the accelerated transport by simple diffusion through the lipid bilayers or through channel-like pores. In the erythrocyte membrane, i.e., the most extensively studied preparation, this “facilitated diffusion” contributes the hypothesis that a membrane-spanning mechanism such as a protein may account for the glucose trans-effect [29] together with other important features including the competition between d-glucose and other sugars [37]. Thus, the term “facilitated diffusion” in this review excludes Na^+^/sugar cotransport.

## 3. The Molecular Identities Mediating Facilitated Diffusion and Na^+^/Sugar Cotransport in Mammals

In the 1980s, glucose transporters that mediate “facilitated diffusion” and Na^+^/sugar cotransport were identified one after another. GLUT1 was identified in human erythrocytes as a facilitated diffusion-type glucose transporter [38]. SGLT1 was then cloned as a Na^+^/sugar cotransporter in the rabbit small intestine [32,39]. During the twentieth century, most GLUTs and SGLTs isoforms had been reported, although further efforts were required to determine the crystal structures [31,32,40,41]. To avoid confusion, the nomenclature for the GLUT family was published by leading investigators in 2002 [42]. In this nomenclature, twelve isoforms are referred to as GLUT1-12 so that they parallel the numbering of sugar transporter gene nomenclature (i.e. SLC2A1-12). 

The member proteins were classified into three categories according to sequence similarities. Class I is the best characterized glucose transporter and comprises GLUT1-4, whereas Class II (GLUT5, 7, 9, 11) are fructose transporters except for GLUT9, which is a urate transporter [31]. Class III comprises GLUT6, 8, 10, and 12, of which physiological roles are only poorly understood [31,43]. One, formerly termed GLUT13, was later designated HMIT, as it is a proton-myoinositol cotransporter. Finally, one formerly designated a pseudogene (chromosome localization 12p13.3) in the nomenclature of 2002 was later termed GLUT14 (SLC2A14) [44]. Because GLUT14 may be duplicated from GLUT3 (94.5% identical to GLUT3), it was categorized in Class I [44,45].

From these considerations, it may be understood why the vast majority of studies on facilitated diffusion-type glucose transport have focused on GLUT1-4 (gene name SLC2A1-4) [31]. These classical glucose transporters show diverse tissue distributions. GLUT1 occurs ubiquitously in erythrocytes and vascular endothelial cells, especially in the brain [38,46]; GLUT2 occurs in liver, small intestine, renal tubules, and pancreatic β-cells (in rodents) [47,48]; GLUT3 occurs in neurons [49]; and GLUT4 occurs in adipose tissues [50] and striated muscle [31,51,52,53]. 

Physiologically, the classical transporters GLUT1-4 may have a common feature in that they transport 2-deoxy-d-glucose (2-DG) [54] and 2-[^18^F]fluoro-2-deoxy-d-glucose (2-FDG) [55] in addition to d-glucose [45,51,52,53,56,57,58]. Moreover, GLUT1, 2, and 4 may transport d-glucosamine (i.e., 2-amino-2-deoxy-d-glucose) as well [59]. This may suggest that classical facilitated diffusion of glucose transport allows entry of d-glucose analogues, in which the hydroxy group at C-2 position is substituted by an amino group or a hydrogen or fluorine atom. This feature may be crucial when developing fluorescence-emitting d-glucose tracers that permeate GLUTs [60]. The nitrogen-substituted structure at the C-2 position of d-glucose is, as with that of d-galactose, physiologically important because the structure is central to glycobiology and related diseases including cancer [61,62].

**Figure 1:**
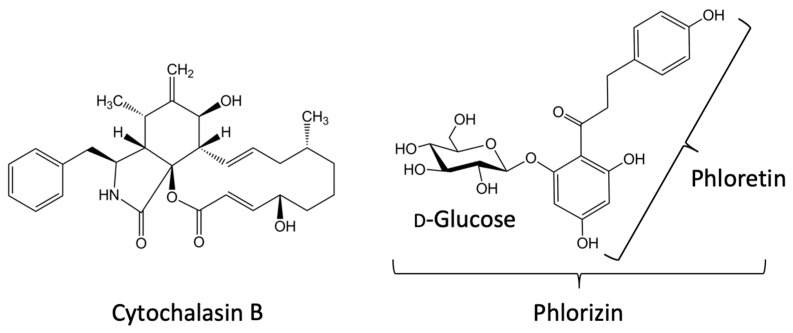
Structures of cytochalasin B, phlorizin, and phloretin.

From the pharmacological point of view, the four classical glucose transporters GLUT1-4 are all inhibited by a small amount of cytochalasin B as well as by phloretin, the aglycone of a major apple polyphenol phlorizin [43,53,63,64,65] (Figure 1). Indeed, cytochalasin B is used to purify the glucose transporter in human erythrocytes (i.e. Class 1) [66]. In contrast, the Class 2 GLUTs, which transport fructose, are insensitive to cytochalasin B [43]. 

For the Na^+^/sugar cotransporter group, six SGLTs (SGLT1-6) have been identified. However, SGLT3 is not a glucose transporter but a glucose sensor. SGLT6, termed also SMIT2, is a sodium-myoinositol cotransporter. Thus, SGLT1, 2, 4, and 5 (gene name SLC5A1, 2, 4, 5) may be the principal Na^+^ and d-glucose co-transporters [32,45].

Of these SGLTs, SGLT1 is primarily expressed in the apical membrane of epithelial cells in the small intestine [39]. SGLT2 operates for the reabsorption of d-glucose in the brush border of renal proximal tubule epithelial cells [67]. However, detailed tissue-distributions of SGLTs and their functional importance have yet to be fully understood [58,68]. 

A remarkable pharmacological feature of glucose uptake by SGLTs is that they are inhibited by phlorizin (Figure 1) and by equalization of the extracellular and intracellular sodium ion concentration, i.e., by an extracellular sodium-free condition [32]. Incidentally, 2-FDG only poorly permeates SGLTs, although 4-[^18^F]fluoro-4-deoxy-d-glucose (4-FDG) does permeate SGLTs [57,58]. Thus, replacement of a hydroxyl group by a fluorine atom at C-2, but not at C-4, position of d-glucose is fatal to d-glucose uptake through SGLTs [69].

## 4. Saturable Transport and Stereo-Preference in Glucose Uptake

Carrier protein-mediated uptake of d-glucose via facilitated diffusion led us to the hypothesis that d-glucose uptake follows Michaelis-Menten saturation kinetics as in enzyme-mediated catalysis [30,36]. The uptake occurs in a manner by which d-glucose in the extracellular space initially binds to the carrier protein for uptake to occur [40,41,70]. Accordingly, saturation of the transport would occur when all of the binding sites of the carrier proteins are occupied by d-glucose. Other free d-glucose has to wait until the site is available (the saturable transport). The binding site for d-glucose is postulated to exist in Na^+^/d-glucose cotransporters (symporters) as well [32,40]. Thus, saturable transport is considered to be an essential feature of facilitated diffusion and Na^+^/d-glucose cotransport. 

Such a strict constraint in d-glucose transport should lead to a marked stereoselectivity for uptake of glucose (the stereo-preference for d-glucose) [30]. Indeed, for Na^+^/d-glucose cotransport, Wright and colleagues have reported from electrophysiological estimation that the relative affinity of l-glucose and that of d-glucose to human SGLT1 differed by over two orders of magnitude, suggesting that l-glucose is a very poor substrate for SGLTs [32]. 

For facilitated diffusion-type transport, evaluating the stereoselectivity is more difficult, since electrophysiological evaluation cannot be directly applied to neutral sugars. In dispersed rat pancreatic islet cells, which consist mostly of β-cells that are thought to express mainly GLUT2, Johnson and colleagues demonstrated approximately 10 times less uptake of l-[1-^3^H]glucose compared with that of 3-*O*-methyl-d-[^3^H]glucose, an unmetabolizable d-glucose analogue [71]. Baldwin and colleagues investigated the stereoselectivity of glucose uptake using phospholipid vesicles, in which a monosaccharide transporter purified from human erythrocytes was reconstituted [66]. These authors reported that uptake of d-[^14^C]glucose, but not that of l-[^3^H]glucose, was markedly inhibited by cytochalasin B. According to the saturable transport hypothesis, this can be interpreted as implying that cytochalasin B interferes with the binding of d-[^14^C]glucose, but not that of l-[^3^H]glucose. Alternatively, the monosaccharide transporter of human erythrocytes has no binding site for l-[^3^H]glucose. In either case, these facilitated diffusion-type transporters have a strong stereo-preference for d-glucose over l-glucose [66].

In human erythrocyte membrane, it might be speculated that the cytochalasin B-inhibitable transporter having a binding site for d- (but not l-) glucose dominates the saturable transport of d-glucose. Indeed, cytochalasin B has been used as a key molecule to identify the carrier protein responsible for facilitated diffusion of glucose transport [30,38,64], despite the deficiency that it binds to the F-actin filament as well [72]. As summarized by Frommer and colleagues, a similar glucose uptake mechanism may be at work in saturable transport across species [28]. That is, the binding of extracellular d-glucose causes a change in the glucose transporter from the outward-facing (exofacial) conformation to the inward-facing (endofacial) one, enabling a movement of d-glucose across the plasma membrane to the intracellular space [28,32,40,41]. Although the mechanism is different, the binding of d-glucose to the transport protein complex is a necessary condition in the bacterial glucose phosphotransferase system as well [34]. Phosphorylation of d-glucose then takes place enzymatically to form d-glucose-6-phosphate (G6P) after entry in mammalian cells and during entry in bacterial cells [28,34,73]. G6P can be further catabolized via well-defined pathways as an energy and/or carbon source [74].

However, it should also be noted that proteins having a more or less similar property might be found if gene screenings were conducted based on sequence homology. The situation would be the same if the molecular search was done according to functional similarity. Indeed, both GLUTs and SGLTs are classified in the major facilitator superfamily, although they belong to distinct categories: facilitated diffusion and Na^+^/sugar cotransport, respectively. Thus, there is a common limitation that both of these transporting systems require the binding of d-glucose to operate.

## 5. Non-Saturable, Non-Stereoselective Uptake of Glucose

In the above-described islet cell preparation by Johnson and colleagues, although it was very small, uptake of l-[1-^3^H]glucose increased with time (up to 60 seconds) [71]. Similarly, in the human erythrocyte monosaccharide transporter-reconstituted vesicles studied by Baldwin and colleagues, entry of l-[^3^H]glucose gradually increased to 60 minutes [66]. Although the initial level of l-[^3^H]glucose uptake was much lower than that of d-[^14^C]glucose, the difference became smaller over time due to a linear increase in the l-[^3^H]glucose uptake compared to a saturating increase in d-[^14^C]glucose uptake [66]. A similar tendency was seen at 5 or 10 minutes after starting the uptake. Interestingly, this component persisted in the presence of cytochalasin B, which also increased linearly with time. It is unclear whether or not these small, but relatively linear increases in the l-[^3^H]glucose uptake detected in two independent preparations were due to artifacts such as a loss of membrane integrity. 

A pharmacological feature common to the facilitated diffusion-type glucose transport in mammals is that the transport is inhibited by phloretin, a low concentration of cytochalasin B, and HgCl_2_ [30] (Figure 1). Indeed, intracellular and extracellular binding sites for phloretin and an intracellular binding site for cytochalasin B have been postulated in GLUT1 structure [38,64,70]. It should be mentioned that any one of these inhibitors can influence membrane transport systems other than facilitated diffusion-type glucose transport. For example, phloretin is a broad-spectrum inhibitor not only for glucose transporters, but also monocarboxylate transporters [75], sodium-dependent vitamin C transporter 1 [45], and aquaporin water channels, which permit entry of a variety of non-charged solutes [76]. Caution should be paid to the use of cytochalasin B, since it may affect F-actin as well [72], and HgCl_2_ inhibits aquaporin water channels including aquaglyceroporins [77,78], which not only water, but also glycerol and urea can permeate [79,80].

In 2007, Conde and colleagues reported that in plant cells, uptake of l-glucose as well as d-glucose occur through a mechanism specifically inhibited by HgCl_2_ [81]. By administering l-[^14^C]glucose or d-[^14^C]glucose to *Olea europaea* (olive) cells, the authors demonstrated non-saturable glucose transport over a concentration range of 0.02 to 100 mM when cultivated in a glucose-sufficient condition (around 2% w/v). Not only d-glucose uptake but also l-glucose uptake increased linearly at the same rate depending on their concentrations [81]. Consistently with the non-saturable nature of glucose transport, no stereo-preference for the uptake of d-glucose over that of l-glucose was detected in olive cells in the glucose-sufficient condition. Based on the ineffectiveness of endocytotic inhibitors and the use of the fluorescent endocytotic indicator FM1-43, they speculated that involvement of endocytotic glucose uptake in non-saturable uptake was unlikely in short-term administration (10 minutes), although it might be involved in more prolonged administrations (14 hours).

In contrast, when the olive cells were cultivated in a glucose-starved condition, saturable transport of glucose uptake was detected [81]. Thus, the saturable or non-saturable mode of transport might depend on the environmental glucose levels. Although the precise molecular mechanism is unknown, the authors speculate that HgCl_2_-inhibitable, non-saturable glucose transport in the olive cells might be mediated by aquaglyceroporin-like channels [81]. Such uptake properties of plant cells might be related to the fact that plants need to adapt to extreme changes in the external sugar concentration [82].

## 6. Evaluating Glucose Uptake in Cancer Cells Using Radiolabeled Tracers

We discussed in the previous section non-saturable, non-stereoselective transport of glucose in a plant cell [81], which might well develop in extremely varying glucose concentrations in the environment [82]. Are these findings relevant to other types of cell? It is interesting to compare the glucose transport system of cancerous cells, which may adapt to low oxygen/nutrient conditions such as that in ascites as well as in oxygen/nutrients-rich blood when metastasized. 

The glucose transport in cancer cells has been investigated by using radiolabeled d-glucose tracers effectively. These tracers include [^14^C]-, or [^3^H]-labeled d-glucose, 2-DG, and 3-*O*-methyl-d-glucose; [^18^F]-labeled 2-FDG and 4-FDG [54,55,66,69,83]. They are particularly useful for quantitatively evaluating d-glucose uptake in living tumor cells, cancerous tissues, tumor-bearing animals, and patients as well as transporter-reconstituted vesicles [54,55,66,69,83]. Detection of the tracers can be done using scintillators [66] for counting the activity of the tracers, radiotracer-sensitive plates for taking a high spatial resolution image [84], and photomultipliers for mapping a spatiotemporal uptake pattern [83]. d-glucose transport across the plasma membrane can then be calculated based on net movement of radiolabeled tracers directly and indirectly with hypothetical kinetic models [66,83].

Enhanced d-glucose uptake in cancer cells has long been a major focus in the field of cancer diagnosis and treatment [9,13,19,85]. Numerous examinations of patients with cancer by positron emission tomography (PET) have demonstrated that cancer cells take in 2-FDG more than non-cancerous cells surrounding the lesion do [55,83,86]. Like d-glucose, 2-FDG enters cancerous cells through GLUTs, and is then phosphorylated to 2-FDG-6-phosphate by hexokinase [83]. However, unlike d-glucose-6-phosphate, no further glycolysis occurs for 2-FDG-6-phosphate, since the hydroxyl group at the C-2 position is lacking in this molecule. As a result, the labeled d-glucose tracer, 2-FDG-6-phosphate, accumulates in the cytosol according to the import/metabolism/export rates of the tracer [83]. Thus, 2-FDG-PET may be one of the best non-invasive imaging techniques for functionally detecting cancer in human patients, despite its poor spatial resolution in the range of a few millimeters and the radiation exposure risk for children and women of reproductive age [83,87,88,89].

Enhanced 2-FDG uptake in cancer cells has been attributed to upregulation of GLUTs as well as glycolysis in the cells [9,83,90,91]. However, there is controversy regarding the upregulation of GLUTs. Positive correlations between GLUT1 protein expression and glucose uptake were reported in 57 cervical carcinomas [92] and 17 ovarian carcinomas [93]. Higashi and colleagues reported a correlation between the standardized uptake value (SUV) of 2-FDG and GLUT1 immunoreactivity in 28 pancreatic cancer patients [94]. In addition, in human sarcoma cells, the tumor suppressor p53, the gene most commonly mutated in human cancer [95,96,97], down-regulates GLUT1 and GLUT4 gene expression [98], consistently with the studies reporting GLUT overexpression. Therefore, GLUTs might be expected to be a suitable target of a drug delivery system (DDS) as well, offering facilitated delivery of chemotherapeutic drugs to cancerous cells [99,100]. However, such an approach has been found to be limited, as will be discussed later.

Given the enhanced expression of GLUTs in some cancerous cells, it may seem curious that an extremely high contrast image of cancer by 2-FDG-PET is sometimes obtained, even though most cells, both cancerous and non-cancerous, take in 2-FDG through GLUT to a greater or lesser extent even when the imaging is conducted in a fasting condition to suppress uptake of the tracer in adipocytes and muscles [101]. After examination of 34 pancreatic lesions, Higashi and colleagues reported that strong GLUT1 expression was detected in 3/6 benign lesions (50%) as well as in 17/28 malignant lesions (61%) [94]. Avril and colleagues reported no relationship between 2-FDG uptake and GLUT1 immunoreactivity in breast cancer [102]. Marom and colleagues reported that neither GLUT1 nor GLUT3 protein levels correlated with 2-FDG uptake in 73 patients with early-stage, non-small cell lung carcinoma [103]. In a recent review, Mayer and colleagues mentioned that GLUT expression and SUV derived from 2-FDG-PET were only moderately associated in various cancers [104]. These studies cast doubt that GLUTs are responsible for enhanced glucose uptake in cancerous cells, although involvement of non-canonical GLUTs such as GLUT12 could not be excluded [105].

## 7. Monitoring Glucose Uptake into Single Cells with Fluorescence-Emitting d-glucose Tracers

As cancerous tissues comprise highly heterogeneous cells [106], it is difficult to analyze uptake pathways of d-glucose at the single cell level by radiolabeled tracers [107]. To more effectively monitor uptake of glucose, use of glucose analogues bearing a fluorophore may represent a possibility. In 1985, Speizer and colleagues reported synthesis of 6-[*N*-(7-nitrobenz-2-oxa-1,3-diazol-4-yl)amino]-6-deoxy-d-glucose (6-NBDG) by reacting 4-chloro-7-nitrobenz-2-oxa-1,3-diazole (NBD-chloride) with 6-amino-6-deoxy-d-glucose [108]. These authors thus made a fluorophore-labeled 6-deoxy-d-glucose analogue bearing NBD via a nitrogen linker at the C-6 position. The NBD group emits fluorescence when attached to a nitrogen atom; 6-NBDG emits green fluorescence at the excitation and emission wavelengths of 470 and 538 nm, respectively. 

Although the NBD group is among the smallest fluorophores available, it is still considerably larger than the glucose molecule. Therefore, it was expected that 6-NBDG would not be carried by the glucose transporter by steric hindrance even if it could access the binding site of the transporter. In fact, 6-NBDG was taken up into human erythrocytes [108]. Moreover, 6-NBDG uptake in the erythrocyte was inhibited by a small amount of cytochalasin B as well as a large amount of 3-*O*-methyl-d-glucose [108]. In addition, uptake of 6-NBDG was inhibited by d-glucose but not by l-glucose [108]. Furthermore, collapsing the Na^+^ gradient had no effect on the uptake [108]. These results strongly suggest that 6-NBDG can be transported by facilitated diffusion-type glucose transporters into human erythrocytes. A major drawback of 6-NBDG is its inability for phosphorylation at the C-6 position of d-glucose moiety because of the attached NBD group.

**Figure 2:**
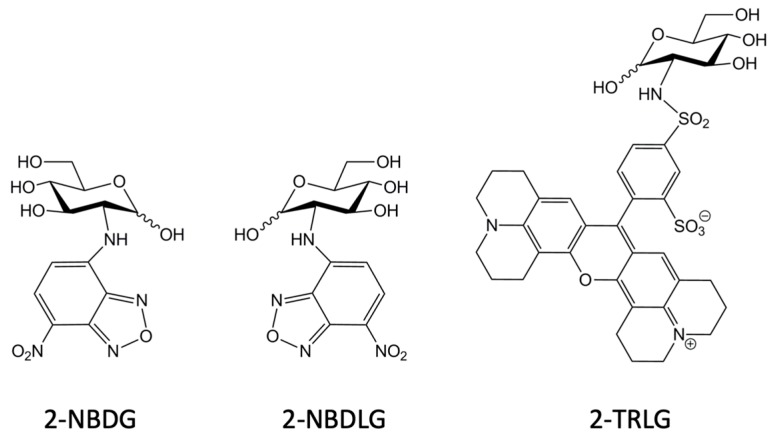
Structures of NBD-conjugated green fluorescence-emitting d-glucose tracer 2-NBDG, NBD-conjugated l-glucose tracer 2-NBDLG (the mirror image isomer of 2-NBDG), and Texas Red-conjugated, red fluorescence-emitting, membrane-impermeable l-glucose analogue 2-TRLG.

In 1996, Matsuoka and colleagues published three consecutive papers about a novel d-glucose tracer [109,110,111]. By reacting NBD-chloride with 2-amino-2-deoxy-d-glucose (d-glucosamine), they synthesized 2-[*N*-(7-nitrobenz-2-oxa-1,3-diazol-4-yl)amino]-2-deoxy-d-glucose (2-NBDG) [109] (Figure 2). Their aim was to count single living microorganisms such as pathogenic bacteria by monitoring not only membrane transport, but also cellular metabolism, i.e., phosphorylation at C-6 position. Thus, 2-NBDG may be regarded as 2-deoxy-d-glucose bearing NBD at C-2 position via a nitrogen linker.

**Figure 3:**
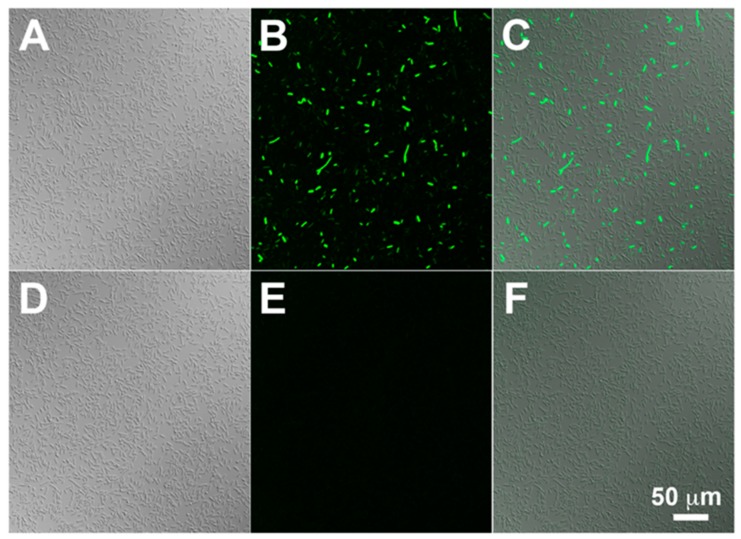
Stereo-preference of living *E. coli* cells for fluorescence-emitting d-glucose tracer 2-NBDG (**A**–**C**) over l-glucose tracer 2-NBDLG (D-F) [112]. (**A**) and (**D**), differential interference contrast images. (**B**) and (**E**), fluorescence images after administration of the fluorescence-emitting D- and L- glucose tracers, respectively. (**C**) and (**F**) are merged images. Images were taken for *E. coli* DH-5α^TM^ cells under the same condition by a confocal microscope (TCS-SP5, Leica) at excitation and emission wavelengths of 488 nm and longer than 500 nm, respectively. The scale bar is common to all panels (Images were taken by Drs. Katsuhiro Nagatomo and Katsuya Yamada, Hirosaki University Graduate School of Medicine).

After incubation with 2-NBDG for 10 minutes, living *Escherichia coli* (*E. coli*) cells emitted green fluorescence with varying intensities (Figure 3A–C), whereas no fluorescence was detected in ethanol-treated dead cells (not shown) [109].

The fluorescence of the cells was markedly reduced by d-glucose, but not by l-glucose, suggesting involvement of a saturable system to which d-glucose, but not l-glucose, can bind [109]. Importantly, 2-NBDG is phosphorylated by the *E. coli* cells, generating 2-NBDG-6-phosphate [110]. 2-NBDG-6-phosphate is then decomposed to a non-fluorescent derivative [110]. Similar uptake of 2-NBDG was detected in living yeast *Candida albicans* cells as well [111].

## 8. Uptake of 2-NBDG into Mammalian Cells through GLUTs and its Application

When Matsuoka’s group published the three consecutive papers, it was unknown whether or not 2-NBDG can monitor d-glucose uptake in mammalian cells. In collaboration with Matsuoka, Yamada and colleagues found that 2-NBDG is taken up into mammalian cells through GLUTs [60]. For this purpose, human GLUT expression vector was transfected into African green monkey kidney fibroblast-like COS-1 cells. These COS-1 cells showed a remarkable increase in fluorescence intensity by 2-NBDG administration compared to mock-transfected cells, regardless of whether GLUT1, 2, or 3 was transfected [60]. The effect of pharmacological inhibitors of glucose transport on 2-NBDG uptake also was examined in mouse insulinoma MIN6 cells [113], which are known to express GLUT2 abundantly and a lesser amount of GLUT1 [60]. The fluorescence intensity of MIN6 cells markedly increased when 2-NBDG was administered for a short period (15 seconds). Both phloretin and a small amount of cytochalasin B (10 μM) strongly inhibited 2-NBDG uptake into MIN6 cells in the presence of d-glucose at a physiological concentration (5.6 mM) [60]. Furthermore, 2-NBDG uptake into MIN6 cells was inhibited by d-glucose in a dose-dependent manner, indicating that 2-NBDG uptake occurred via saturable transport [60].

These results are consistent with the hypothesis that 2-NBDG is taken up into mammalian cells through GLUTs. Indeed, the 2-NBDG uptake in MIN6 cells also occurred in a time, concentration, and temperature-dependent manner [60]. Eadie-Hofstee transformation of the relationship between the concentration of 2-NBDG and the initial velocity of its uptake into MIN6 cells resulted in a non-linear curve with two kinetic components, Km values of 13.3 mM and 1.6 mM [60]. Pancreatic islets as well as cultured pancreatic β-cells express GLUT1 at low levels, although GLUT2 is much more abundant [114,115]. Km values reported for GLUT2 and GLUT1 in dispersed rat pancreatic islet cells analyzed by 3-*O*-methyl-d-glucose were 17 mM and 1.4 mM, respectively [71]. As MIN6 cells express not only GLUT2 but also GLUT1 at a very low level [113], high Km and low Km may correspond to the affinities of 2-NBDG for GLUT2 and GLUT1, respectively. Pancreatic islet cells acutely dissociated from rats were also tested, and demonstrated that GLUT2-expressing β-cells, which respond to high glucose stimulation, took up abundant 2-NBDG, while much less uptake was detected in GLUT1-expressing α-cells, which show no response to high glucose stimulation [60]. 

Around the same time, Lloyd and colleagues synthesized 2-NBDG according to the Matsuoka protocol, and administered it to vascular smooth muscle cells of pigs [116]. They found a time-dependent increase in 2-NBDG uptake that was inhibited by d-glucose and not by l-glucose [116]. When searching PubMed for 2-NBDG, 300 publications presently come up; these include application to a wide variety of tissues and cells such as skeletal muscle cells [117], cardiomyocytes [118,119], vascular endothelial cells [120], enterocytes [121,122], pancreatic cells [123,124,125], neurons and astrocytes [11,12,126,127,128,129,130], cochlea cells [131], retinal cells [132], sperm and ovary cells [133], lymphocytes [134,135], and pluripotent stem cells [136]. The most epoch-making of these would be the finding of gap junction-mediated, glucose trafficking in the brain [11,12].

In addition, several groups have shown that 2-NBDG is useful for monitoring aberrant cellular uptake of d-glucose in tumors in vitro and in vivo [137,138,139,140,141,142,143,144,145]. 2-NBDG has been effectively applied to clinical specimens as well such as biopsy tissues obtained from patients with oral cancer [146,147], esophagus cancer [148], head and neck cancer [149], breast cancer [150], colorectal cancer [151], and for metabolic phenotyping or screening of cancerous cells in pleural effusion or peripheral blood obtained from lung cancer patients [152,153]. 

## 9. Development of d-glucose Analogues that Emit Fluorescence of Various Wavelengths and Bear Cytotoxic Substituents Targeting Cancer

**Figure 4:**
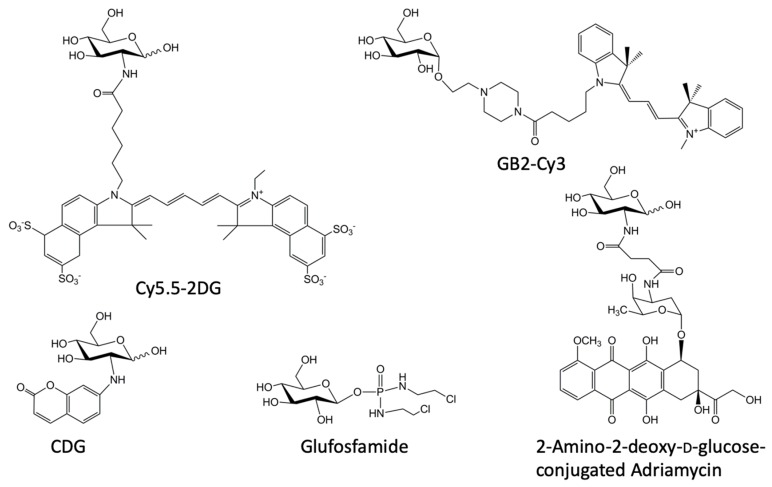
Structures of various d-glucose analogues.

The successful monitoring of d-glucose uptake by 2-NBDG especially in tumors has stimulated numerous attempts to develop d-glucose analogues emitting brighter and more tissue-penetrable fluorescence than 2-NBDG, aiming at detecting fluorescence from deep in cancerous tissues [154]. 2-NBDG has a molecular weight of 342 and emits green to yellow fluorescence at the maximum emission wavelength (Em) of 540–550 nm [155]. If an analogue having longer Em is used, tissue-penetrability of the fluorescence would increase, but the molecular weight would also increase, making it more difficult for the analogue to be taken up through the glucose transporters [156]. Shown here are representative analogues having Em longer than that of 2-NBDG: near-infrared (NIR) dyes Cy5.5-2DG [157] and IRDye 800CW 2-DG [158] and orange fluorescence-emitting GB2-Cy3 [159] (Figure 4).

Cy5.5-2DG (Em, 695 nm) shows a high tissue-penetration property and accumulates in various tumor cell lines when administered in vivo as well as in vitro [157]. However, the uptake was not competitively inhibited by a large dose of d-glucose, indicating that GLUTs are not the transporter system responsible for the uptake [157]. IRDye 800CW 2-DG (Em, 789 nm, detailed structure not disclosed) is made by LI-COR Biosciences (Lincoln, Nebr., USA) [158]. When MDA-MB-231 cells are incubated with IRDye 800CW 2-DG for an hour, the fluorescence of the cells increases [158]. This increase in fluorescence, unlike that in Cy5.5-2DG, was attenuated by d-glucose in a dose-dependent manner [158]. Accumulation of fluorescence was detected in tumor-bearing mice by repeated intravenous injections [158].

GB2-Cy3 (Em, 555 nm) was synthesized by attaching Cy3, a cyanine dye much smaller than Cy5.5, to C-1 position of d-glucose via a long linker [159] (Figure 4). Although GB2-Cy3 has relatively large molecular weight (M.W., 718), it accumulates in several cell lines when administered for 30 minutes [159,160]. This accumulation is inhibited by a large amount of d-glucose, which is consistent with the hypothesis that a mechanism requiring binding of d-glucose such as GLUTs is involved [159,160]. The same group of authors mentioned that the accumulation of GB2-Cy3 in muscle cells was increased after insulin treatment and that this increase was blocked by wortmannin [154,161]. Wortmannin is known to inhibit massive endocytosis [162]. Indeed, an incubation with d-glucose analogues including 2-NBDG for a long period may cause an endocytotic process that can be blocked by wortmannin [81]. Thus, detailed kinetic analyses of the uptake for a short and long incubation period are awaited in combination with pharmacological examinations to exclude the possibility that the d-glucose analogues IRDye 800CW 2-DG and GB2-Cy3 accumulated in the cells due to internalization of a membrane protein that binds d-glucose and its analogues [60,81,155].

An alternative approach would be to use d-glucose analogues having shorter Em for facilitating their uptake through GLUTs [156]. Even though tissue penetrability was poor, a higher specificity for GLUTs might be obtained if designed adequately. 2-Deoxy-2-(2-oxo-2*H*-chromen-7-yl)amino-d-glucose (CDG; Em, 455 nm, M.W. 323) was developed by such a strategy [156] (Figure 4). CDG is a small d-glucose analogue with a conjugated coumarin at the C-2 position. As reported by Supuran and colleagues, coumarin interacts with specific carbonic anhydrases IX and XII that are expressed on the plasma membrane of tumor cells [163]. However, when administered to MIN6 cells, unlike coumarin alone and many of its analogues, CDG was taken up immediately into the cells with pharmacological properties very similar to those of 2-NBDG, suggesting GLUT-mediated uptake [156,164]. 

When considering clinical application of fluorescent d-glucose analogues, it is worth mentioning that most tumors in the digestive tract first appear as anomalies in the luminal surface [165,166]. Therefore, short wavelength fluorophores such as yellow, green, and even blue are potentially applicable to endoscopic examination. Indeed, successful evaluations of clinical specimens by 2-NBDG have been reported [146,147,148,149,150]. Another possibility is to use 2-NBDG as a marker for metabolic phenotyping of body fluid [152,153,167].

As discussed previously, some investigators have thought that GLUTs could be a target of a DDS, providing enhanced glucose uptake in cancerous cells due to GLUT overexpression in these cells [168]. Thus, many d-glucose analogues bearing cytotoxic agents have been synthesized, hoping for enhancing delivery of the cytotoxic effect to cancer via GLUTs [168] (Figure 4). Wiessler and colleagues synthesized glufosfamide by attaching the well-known DNA-alkylating agent ifosfamide (isophosphoramide mustard) to C-1 position of d-glucose [169]. Interestingly, the effect of glufosfamide was inhibited by phloretin and phlorizin (Figure 1), consistent with the idea that the uptake mechanism of this d-glucose analogue involves saturable processes like GLUTs and SGLTs [169]. Glycosylation of the cytotoxic agent facilitated the efficacy of the drug delivery in rats and mice as well, although it was readily hydrolyzed due to the C-1 conjugation [169,170]. 

Clinical trials in patients with solid tumors of various origins suggest that intravenously administered glufosfamide may have some slowing effects on cancer progression, although it caused a toxicity to renal processing [171]. If d-glucose conjugated with a cytotoxic agent is easily cleaved at its conjugation site, the anti-cancer effect would be similar to that of the agent alone, even though the water-solubility was improved by glycosylation. However, if this is not the case as in 2-amino-2-deoxy-d-glucose conjugate of adriamycin (doxorubicin) [172] (Figure 4), an anti-cancer drug targeting GLUTs would affect normal cells as well as cancerous cells [168]. This is an essential limitation of d-glucose analogues.

## 10. Development of a Fluorescence-Emitting l-glucose Tracer as a Control Substrate for 2-NBDG

Although 2-NBDG has been used in an increasing number of studies, quantification of the uptake was sometimes difficult, as fluorescence intensity is an arbitrary measure [155]. This could be particularly problematic when the uptake is used for cancer staging or for delicate cells such as neurons having easily damaged membrane integrity [24,155].

Glucose may adopt either d-, or its mirror-image l-, conformations [173,174]. Cells do not catabolize [23] or take up l-glucose in an amount worth mentioning, at least through saturable transport [32,66,71,175], except in the case of some Gram-negative bacteria [25,27] and plants under certain conditions [81]. Thus, an l-glucose analogue in which the hydroxyl group at C-2 position is substituted by an NBD group with a nitrogen-linker would be an ideal control substrate against 2-NBDG (Figure 2); if cells of interest took up the l-glucose tracer as well as 2-NBDG, the uptake would not be mediated by saturable glucose transporters like GLUTs and SGLTs, which require stereo-selective binding of glucose moiety to the binding site for their operation.

By reacting NBD-Cl with l-glucosamine, the mirror-image isomer of 2-NBDG was developed as the first fluorescence-emitting tracer of l-glucose [176]. Since l-glucosamine was not commercially available, it was newly synthesized from l-mannose in 10 steps [176]. This molecule, 2-[*N*-(7-nitrobenz-2-oxa-1,3-diazol-4-yl)amino]-2-deoxy-l-glucose, is referred to as 2-NBDLG [112,176] (Figure 2). When administered to *E. coli* DH5α, 2-NBDLG caused no increase in the fluorescence in the cells, whereas 2-NBDG caused increase in the fluorescence with variable intensity [112] (Figure 3). Since only the glucose moiety differs between 2-NBDLG and 2-NBDG, it is likely that uptake of 2-NBDG into the *E. coli* DH5α cells was mediated by the saturable transport that differentiates between d-glucose and l-glucose.

## 11. Specific Uptake of a Fluorescence-Emitting l-glucose Tracer in Tumor Spheroids

The efficacy of 2-NBDLG as a control substrate was examined in mammalian cells as well. The initial study was conducted using non-cultured neurons acutely dissociated from the adult mouse midbrain (the substantia nigra pars reticulata) [177]. Although clear cytosolic increase in the fluorescence was detected by administration of 2-NBDG (100 μM) for 1 minute, only negligible fluorescence could be detected after similarly administered 2-NBDLG [177]. However, statistical comparison was not easy, as these acutely dissociated neurons comprised a mixture of a large number of spontaneously active (i.e., energy-demanding), but delicate GABAergic neurons and a small number of non-active, but tough dopaminergic neurons [178]. 

Thus, to analyze a large number of cells simultaneously, mouse insulinoma MIN6 cells (5–10 passages) [113] were used [179]. After 3–6 days in vitro (DIV), superfusion of MIN6 cells with 100 μM 2-NBDG for three minutes followed by washout increased the cellular fluorescence with varying intensities among cells, whereas no such increase in the fluorescence was detected by similarly applying the same amount of 2-NBDLG [179]. 

For statistical comparison, MIN6 cells were seeded on a 96-well microplate, and the average fluorescence was then measured for each well by a microplate reader [179]. Since MIN6 cells at earlier passages tend to aggregate three-dimensionally while leaving a large space between cells (i.e., no fluorescence), measurements were conducted at 10–14 DIV when enough intensity of fluorescence was obtained. To our surprise, the ratio of the net increase in the fluorescence before and after administration of 2-NBDLG to that of 2-NBDG was 44.9 ± 1.7% on average [179]. Measurements were conducted in the presence of a physiological concentration (5.6 mM) of d-glucose and 100 μM carbenoxolone to exclude non-specific uptake through gap junctions/hemichannels.

Interestingly, when cultured for over 10 days, MIN6 cells grown at the edge of culture dishes or glass cover slips tended to form thick spheroids consisting of tightly accumulating cells [179]. Confocal microscopic examination revealed that 2-NBDLG was taken up into some of such spheroids, particularly when they contained cells showing a remarkable cellular and nuclear heterogeneity, while no such uptake was detected in spheroids consisting of small cells with homogeneous nuclei [179,180]. This is of special interest because cellular and nuclear heterogeneity is among the critical features of malignancy [181,182,183].

**Figure 5:**
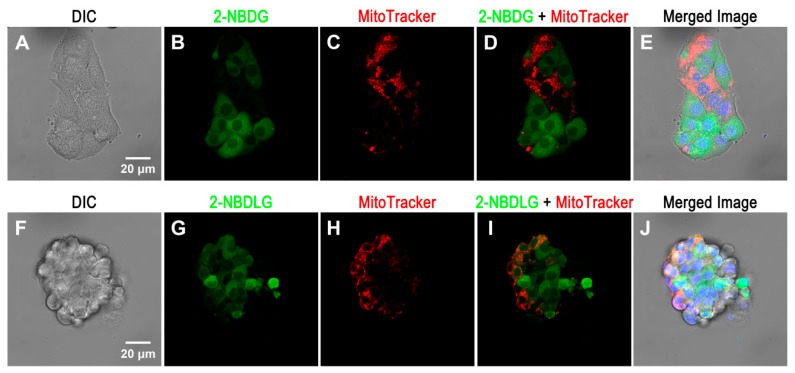
Representative confocal microscopic images of mouse insulinoma MIN6 cell aggregates exposed for a short period to Krebs-Ringer buffer solution containing either 2-NBDG (upper panels) or 2-NBDLG (lower panels). On 7 DIV, uptake of 2-NBDG (**A**–**E**) or 2-NBDLG (**F**–**J**) was examined simultaneously with live-staining by 4’,6-diamidino-2-phenylindole (DAPI) (blue, for nuclear staining) and MitoTracker Deep Red FM (red, a mitochondrial membrane potential marker). (**A**) and (**F**) represent differential interference contrast (DIC) images. (**B**) and (**G**) represent fluorescence images of MIN6 cells taking up 2-NBDG (B, green) and 2-NBDLG (G, green), respectively. (**C**) and (**H**) represent MitoTracker images (red). (**D**) and (**I**) represent overlays of (**B**) and (**C**), and those of (**G**) and (**H**), respectively. (**E**) and (**J**) are merged images. (**A**–**E**) show cells exhibiting a stronger 2-NBDG uptake and lower mitochondrial membrane potential, and vice versa. At this culture stage, some MIN6 cells formed a small spheroid, in which varying 2-NBDLG uptake was detected (Images were taken by Shota Takigawa and Katsuya Yamada, Hirosaki University Graduate School of Medicine).

Moreover, on 7 DIV, the stage when most of the cells formed only thin aggregates or immature spheroids, a preliminary (unpublished) experiment in our laboratory indicated an interesting relationship between 2-NBDG uptake and mitochondrial activities evaluated by a probe reflecting the mitochondrial membrane potential (MitoTracker Deep Red FM, ThermoFisher Scientific, Waltham, MA, USA) (Figure 5A). In this MIN6 cell aggregate, cells showing higher 2-NBDG uptake appeared to exhibit lower mitochondrial membrane potential, while cells showing higher mitochondrial membrane potential exhibited lower 2-NBDG uptake (Figure 5B–E). This is truly impressive, as Otto Warburg himself noted that cancer cells may change their pathways for glucose metabolism from the well-known mitochondria-mediated ones to those known for glucose fermentation in microorganisms (Warburg Effect) [19].

Furthermore, at the same culture stage, some MIN6 cells in an immature spheroid (Figure 5F) had already started to take up 2-NBDLG with varying intensities (Figure 5G). While these MIN6 cells taking up abundant 2-NBDLG demonstrated lower mitochondrial membrane potential (Figure 5G–I), not all of MIN6 cells showing lower mitochondrial membrane potential took up 2-NBDLG (Figure 5I). While lowered mitochondrial membrane potential is not a sufficient condition for uptake of 2-NBDLG in these MIN6 cells, 2-NBDLG uptake in these cells implies that the cells showing lowered mitochondrial membrane potential are heterogeneous (Figure 5G–J).

## 12. The Mechanism of 2-NBDLG Uptake into MIN6 Cells

As was visualized by the fluorescence-emitting d- and l-glucose tracers, it is likely that MIN6 cells grown over a week comprise mostly heterogeneous populations of cells. To elucidate the mechanisms that regulate the 2-NBDLG uptake, Sasaki and colleagues cultured MIN6 cells for 10-14 days in 96-well microplates [179]. The first question was whether or not glucose transporters mediate 2-NBDLG uptake. 2-NBDG was used as a control. A small amount of cytochalasin B (10 μM), an inhibitor of facilitated diffusion (Figure 1), significantly suppressed 2-NBDG uptake in the MIN6 cells, although a considerable amount of the uptake remained unaffected even in the presence of cytochalasin B [179]. 

In contrast, 2-NBDLG uptake, which was examined simultaneously in the same 96-well plates, largely persisted in the presence of cytochalasin B, implying that most of the 2-NBDLG uptake was mediated by mechanisms other than facilitated diffusion as in GLUTs [179]. 

Involvement of Na^+^/sugar cotransporters is unlikely. In fact, neither 2-NBDLG nor 2-NBDG uptake into MIN6 cells was affected by extracellular Na^+^-free condition [179]. These results suggest that a mechanism other than saturable transport mediates the 2-NBDLG uptake in MIN6 cells [179]. Consistently, no competitive inhibition by a large amount of d-glucose or l-glucose was detected in 2-NBDLG uptake into the MIN6 cells [179]. These results are in contrast to the finding that a large amount of d-glucose, but not l-glucose, suppressed the 2-NBDG uptake significantly [179].

**Figure 6:**
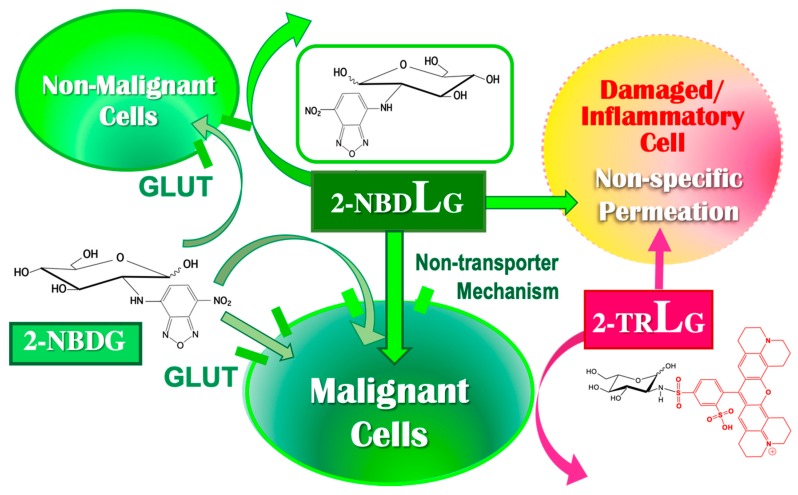
Schematic representation of uptake of fluorescence-emitting d- and l-glucose tracers. Although 2-NBDG enters both non-malignant and malignant cells, 2-NBDLG enters malignant cells only, when plasma membrane integrity is preserved. 2-TRLG is used to identify cells in which 2-NBDLG is taken up due to a loss of membrane integrity. Modified from Yamada, K. Biol. Pharm. Bull. Vol. 41 No. 10. Front cover (Copyright 2018 The Pharmaceutical Society of Japan).

A clue to understand the mechanism underlying 2-NBDLG uptake came from a study in plant cells. Conde and colleagues reported HgCl_2_ as an effective inhibitor of the non-saturable component of 2-NBDG uptake into olive cells [81]. They speculated that the HgCl_2_-inhibitable uptake of 2-NBDG in plant cells might be due to aquaglyceroporin-like channels [81,82]. Aquaglycreroporins are blocked by HgCl_2_ and phloretin [76] (Figure 1). The experimental results are clear. Phloretin totally abolished the uptake of 2-NBDLG in MIN6 cells [179]. Moreover, phloretin completely blocked the 2-NBDG uptake that remained in the presence of cytochalasin B **[179]**, indicating that phloretin is capable of blocking both the uptake of the l-glucose tracer as well as the d-glucose tracer non-stereoselectively. Similar inhibitions by cytochalasin B and phloretin against uptake in MIN6 cells were detected for CDG (Figure 4) and CLG, which are the blue fluorescence-emitting, coumarin-conjugated d-glucose tracer and its mirror image isomer, respectively [156,164]. Incidentally, NBD alone (neither NBD-NH_2_ nor NBD-Cl) elicited no detectable fluorescence in MIN6 cells, indicating that the glucose moiety is required for the uptake to occur. As described previously, phloretin is a broad-spectrum inhibitor of transporters and channels, including GLUTs, monocarboxylate transporters, Na^+^-dependent vitamin C transporter 1, and aquaporins. Thus, non-GLUT/non-SGLT, non-saturable, possibly channel-like mechanisms might well mediate 2-NBDLG uptake into MIN6 cells [24,179] (Figure 6).

**Figure 7:**
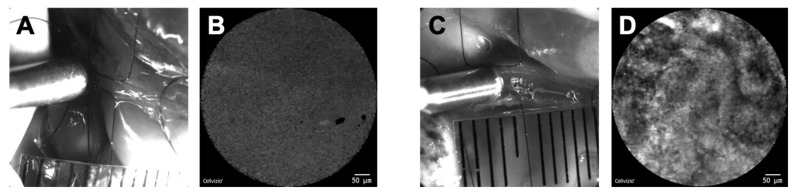
Confocal endomicroscopic imaging of the bile duct lumen of hamster. Administration of fLGs for 3 minutes followed by washout produced a homogeneous fluorescence pattern in normal bile duct lumen of a healthy hamster (**A**,**B**), whereas a highly heterogeneous pattern was detected in the lumen of a carcinogen BOP-administered hamster (**C**,**D**) at the same condition [184]. (**A**) and (**C**) represent the position of the confocal probe at the time of imaging. (**B**) and (**D**) represent corresponding confocal endomicroscopic images of the bile duct lumen. Scale bars in (**B**) and (**D**) represent 50 μm.

For 2-NBDG uptake into MIN6 cells at over 10 DIV, there are two components: saturable transport sensitive to cytochalasin B and non-saturable transport insensitive to cytochalasin B (Figure 6). The cytochalasin B-insensitive component of 2-NBDG uptake was abolished by phloretin [179], suggesting that this component is non-stereoselective. It would be of interest to identify the molecular mechanisms underlying cytochalasin B-insensitive, phloretin-inhibitable transport by using 2-NBDLG. 2-NBDLG uptake has been detected not only in cultured tumor cells, but also in in vivo carcinomas including those developed in hamster bile duct [184] (Figure 7). 

This cholangiocarcinoma was induced by an injection of the carcinogen *N*-nitrosobis(2-oxopropyl)amine (BOP) for 9 weeks combined with cholecystoduodenostomy (surgical anastomosis of the gallbladder and the duodenum) with ligation of the extrahepatic bile duct in the distal end of the common duct. Confocal endomicroscopic imaging of the bile duct lumen conducted in vivo followed by pathological evaluation of the imaged sites revealed statistically significant correlation between the fLG uptake patterns and the histopathological grades [180,184].

In actual applications of the fluorescent tracers, the membrane condition of delicate cells like neurons and those around the tumor is not always healthy [185,186]. Indeed, both 2-NBDLG and 2-NBDG can enter membrane-damaged cells irrespective of whether they are tumor or non-tumor cells [177] (Figure 6). To identify non-specific entry into such damaged cells, an l-glucose analogue bearing the large red fluorophore Texas-Red (sulforhodamine 101 acid) at C-2 position was developed [177,179]. This molecule, Texas Red-coupled 2-deoxy-l-glucose, is referred to as 2-TRLG (Figure 2 and Figure 6). Using 2-TRLG in combination with 2-NBDLG or 2-NBDG, non-specific entry into membrane-damaged cells was identified by 2-TRLG entry, and then excluded from the uptake analysis [177,179]. Unlike dead cell markers such as propidium iodide, 2-TRLG is sensitive to slight changes in the membrane state of damaged and dying but not dead cells, marking such cells in color: yellow, orange, or red depending on time and the membrane state when administered with 2-NBDLG or 2-NBDG [24,177,179] (Figure 6). 

Regarding the toxicity of fLGs, both 2-NBDLG and 2-TRLG exhibited no mutagenic potential in the bacterial reverse mutation test (the Ames test) according to Good Laboratory Practice regulations. No appreciable toxicity was detected in expanded single-dose oral toxicity tests as well according to the same regulations [24]. This is rather surprising, considering that fluorescent analogues are generally toxic to organisms and that only a few fluorescent tracers such as fluorescein and indocyanine green have been routinely used in clinical practice over decades. The l-glucose moiety that is common to these fLGs should contribute to their low toxicity. These results reinforced the fact that only minimum uptake of 2-NBDLG was detected in normal cells as described. Application of fLGs to clinical specimens and survey of patient outcomes are ongoing. Completion of the survey is awaited [24]. 

The newest 8th edition of the American Joint Committee on Cancer staging manual has changed from solely according to anatomic information as the TNM classification, which classifies tumors by size, lymph node metastasis, and distal metastasis, to a prognostic staging system integrating functional anomalies in consideration of the remarkable progress of modern molecular biology and the importance of biologic markers [187]. Fluorescent tracers that reflect metabolic states of single living cells such as fLGs are promising candidate molecules to improve the accuracy of cancer diagnosis. 

## 13. Future Perspectives

l-glucose is a sugar that is rarely found in Nature. Even when administered, mammals take it up only minimally [188]. Furthermore, even if it were taken up into cells, it could not be metabolized [23]. As such, l-glucose has long been considered useless, whereas d-glucose is what most, if not all, organisms crave. However, it is curious that l-glucose tastes no less sweet than d-glucose, and the l-glucose molecule is the mirror image of the d-glucose molecule. Among amino acids, d-amino acids were long considered to be non-functional and to occur rarely in Nature. However, increasing evidence suggests that d-amino acids are present not only in microorganisms [189,190] and plants [191], but also in mammals in a considerable amount, exerting important roles related to mental health and age-related disorders including cancer [189,192,193,194,195,196].

Interestingly, Shimizu and colleagues found an l-glucose catabolic pathway in the soil bacterium *Paracoccus* sp. 43P [27]. l-glucose was taken up in the strain, then was oxidized to l-gluconate, to l-glucono-1,5-lactone, then eventually catabolized to pyruvate and glyceraldehyde 3-phosphate through an L/D conversion at the C-5 position [27]. If the stereo-preference for the sugars in living organisms is inversely correlated with that for the amino acid [197], then the functional significance of l-glucose might well be revealed in near future. 

Besides fluorescence-emitting glucose tracers, the use of a genetically encoded, fluorescence resonance energy transfer (FRET) biosensor is an elegant technique to measure glucose utilization as well as uptake [198]. Using FRET biosensors, Frommer’s group discovered a new class of glucose transporters in plants, the SWEET superfamily [199]. The FRET biosensors may be applicable to a wide range of cellular processes [200], although this type of approach requires precise molecular identification of the target proteins in addition to accurate knowledge of associated cellular events that may affect the energy transfer [201] and molecular information to narrow down the condition when searching genes of the transporting system [198]. Information regarding actual movement of glucose or its analogues as well as changes in the transport proteins would provide better understanding of the whole picture of glucose transport in cells.

## 14. Conclusions

Glucose may be the most versatile energy/carbon source for living beings. Nevertheless, our knowledge of glucose transport is still limited, especially when energy is highly demanded as in tumorigenesis. In the present review, we focused specifically on three-dimensionally accumulated tumor cells showing nuclear heterogeneity and aberrant uptake of fluorescence-emitting tracers of l-glucose through non-stereoselective, possibly non-transporter-mediated, mechanisms [179,180,184]. 

Regarding malignant tumor cells, we hypothesize that not only metabolic flexibility as mentioned by Otto Warburg, but also changes in the fuel uptake system in the plasma membrane as well as the stereoselectivity for the fuel itself may occur in concert to adapt to the harsh demand/supply conditions characteristic of the microenvironments of individual tumor cells.

It is particularly challenging to evaluate the glucose uptake in three-dimensionally assembled, mutually-communicating, highly heterogeneous cell aggregates, where each cell may use functionally divergent uptake mechanisms, either simultaneously or separately even in single cells [49,68,202,203]. Use of fluorescence-emitting l-glucose tracers is a unique method to identify and characterize cancerous cells among such cell aggregates. Compared to the use of specific gene information or antibodies for specific cancers, such glucose tracers may have a wider applicability as shown by the 2-FDG-PET. For better understanding of fuel uptake and utilization, it would be effective to combine information obtained by divergent approaches including not only changes in the proteins that mediate the transport, but also changes in the fuel itself that is transported.
